# The SSAI fully supports the suspension of hydroxyethyl‐starch solutions commissioned by the European Medicines Agency

**DOI:** 10.1111/aas.13120

**Published:** 2018-04-16

**Authors:** J. H. Laake, T. I. Tønnessen, M. S. Chew, M. Lipcsey, H. Hjelmqvist, E. Wilkman, V. Pettilä, J. Hoffmann‐Petersen, M. H. Møller, the Board of the Scandinavian Society of Anaesthesiology Intensive Care Medicine (SSAI)

**Affiliations:** ^1^ Department of Anaesthesiology Division of Critical Care and Emergencies Oslo University Hospital Oslo Norway; ^2^ Department of Anaesthesia and Intensive Care Medical and Health Sciences Linköping University Linköping Sweden; ^3^ Hedenstierna laboratory Department of Surgical Sciences Anaesthesiology and Intensive Care CIRRUS Uppsala University Hospital Uppsala Sweden; ^4^ School of Medical Sciences Department of Anaesthesia and Intensive Care Örebro University and University Hospital Örebro Sweden; ^5^ Department of Anaesthesiology, Intensive Care and Pain Medicine University of Helsinki and Helsinki University Hospital Helsinki Finland; ^6^ Department of Anaesthesiology and Intensive Care Odense University Hospital Odense Denmark; ^7^ Department of Intensive Care Copenhagen University Hospital Rigshospitalet Copenhagen Denmark

The Coordination Group for Mutual Recognition and Decentralised Procedures (CMDh) of the European Medicines Agency (EMA) has recently endorsed the recommendation by EMA's Pharmacovigilance Risk Assessment Committee (PRAC) to suspend the marketing authorisations of hydroxyethyl‐starch (HES) solutions across the European Union, as well as in Iceland, Liechtenstein and Norway.^1^ This has prompted a response from the president of the German Society of Anaesthesiology and Intensive Care Medicine (DGAI) who has invited national anaesthesia societies across Europe to endorse a letter to the EMA and to the European Commission, denouncing the suspension of HES (Prof. Dr. med. Bernhard Zwißler President of the DGAI, personal communication).

In this paper, written on behalf of the Board of The Scandinavian Society of Anaesthesiology and Intensive Care Medicine (SSAI) and the national societies of Iceland, Denmark, Finland, Sweden and Norway, we explain why the SSAI fully supports the recommendation to suspend the marketing authorisation of HES solutions.

HES solutions are intravenous fluid products (colloids) that, until recently, were commonly used in clinical practice.^2^ HES solutions have, however, been found to be associated with adverse outcomes for patients, including acute kidney injury^3^, ^4^, ^5^ and increased risk of bleeding.^6^ In a recently published, high‐quality systematic review of 42 randomised clinical trials (11 399 patients), HES products were associated with a 59% increased risk of acute kidney injury, and a 32% increased risk of renal replacement therapy compared to other intravenous fluids.^7^ Importantly, these adverse events were confirmed in all patient populations independent of the dosage and type of HES used. Moreover, a 2013 Cochrane review of randomised clinical trials found no evidence that resuscitation with colloids improved outcome in surgical patients, including patients with burns and trauma.^8^ Importantly, patients who received HES solutions had increased mortality. These high‐quality data underline that use of HES as volume replacement in patient populations relevant to anaesthetists and intensivists, including surgical patients and critically ill patients, is associated with harm, including acute kidney injury, bleeding and death.

Following the raised concerns about the safety of HES, some research groups and societies have continued to recommend use of HES solutions based on studies with serious methodological flaws and with significant financial and academic conflict of interests.^9^, ^10^

Recently, it was pointed out that an earlier decision by the EMA (2013) to restrict the use of HES to non‐septic patients with haemorrhagic shock was not being followed in many countries, and that HES solutions continue to be used in populations at high risk, eg, postpartum haemorrhage.^11^ In an appeal to the World Health Organization, the authors called for a global ban of HES. If *primum non nocere (*first do no harm) is to be our guiding principle, this is the logical next step.

In summary, there is high‐quality evidence that use of HES solutions as volume replacement in critically ill patients in general, including surgical patients and ICU patients increases the risk of adverse outcomes, including acute kidney injury, bleeding and death.^5^, ^7^, ^8^ Also, there are no valid data indicating that HES solutions are superior to crystalloid solutions in any population. Therefore, the use of HES needs to stop immediately. Thus, the SSAI and the Nordic national anaesthesia and intensive care societies fully support the suspension of HES solutions as suggested by the EMA.

## CONFLICT OF INTEREST

None.
